# Global risk of invasion by *Bactrocera zonata*: Implications on horticultural crop production under changing climatic conditions

**DOI:** 10.1371/journal.pone.0243047

**Published:** 2020-12-23

**Authors:** Kumbirai M. Zingore, George Sithole, Elfatih M. Abdel-Rahman, Samira A. Mohamed, Sunday Ekesi, Chrysantus M. Tanga, Mohammed E. E. Mahmoud

**Affiliations:** 1 International Centre of Insect Physiology and Ecology (*icipe*), Nairobi, Kenya; 2 Geomatics Division, School of Architecture, Planning and Geomatics, University of Cape Town, Rondebosch, South Africa; 3 Department of Agronomy, Faculty of Agriculture, University of Khartoum, Khartoum North, Sudan; 4 Agricultural Research Corporation, Wad Medan, Sudan; Sichuan University, CHINA

## Abstract

The peach fruit fly *Bactrocera zonata* (Saunders) (Diptera: Tephritidae) is an important invasive species causing substantial losses to the horticulture industry worldwide. Despite the severe economic impact caused by this pest in its native and invaded range, information on its potential range expansion under changing climate remains largely unknown. In this study, we employed maximum entropy (MaxEnt) modeling approach to predict the global potential climatic suitability of *B*. *zonata* under current climate and four Representative Concentration Pathways (RCPs) for the year 2050. Outputs from MaxEnt were merged with Spatial Production Allocation Model. A natural dispersal model using Gaussian dispersal kernel was developed. The Areas Under Curves generated by MaxEnt were greater than 0.92 for both current and future climate change scenarios, indicating satisfactory performances of the models. Mean temperature of the coldest quarter, precipitation of driest month and temperature seasonality significantly influenced the potential establishment of *B*. *zonata*. The models indicated high climatic suitability in tropical and subtropical areas in Asia and Africa, where the species has already been recorded. Suitable areas were predicted in West, East and Central Africa and to a lesser extent in Central and South America. Future climatic scenarios models, RCP 4.5 and 8.5 show significant potential range expansion of *B*. *zonata* in Western Sahara, while RCP 4.5 highlighted expansion in Southern Africa. Contrarily, RCP 2.6 showed considerable decrease in *B*. *zonata* range expansion in Central, East and West Africa. There was increased climatic suitability of *B*. *zonata* in Egypt and Middle East under RCP 6.0. The dispersal model revealed that *B*. *zonata* could spread widely within its vicinity with decreasing infestation rates away from the source points. Our findings can help to guide biosecurity agencies in decision-making and serve as an early warning tool to safeguard against the pest invasion into unaffected areas.

## Introduction

Global pest invasions promoted by numerous pathways availed by growing travel and world trade have increased in the recent years impacting on ecosystems, economic activities and human welfare [1–3]. Tephritid fruit flies of the genus *Bactrocera* have particularly caused great concern due to the magnitude of damage they inflict. The mated female fruit flies lay eggs in ripening fruit, followed by larvae and other opportunist secondary microorganisms feeding on the fruit pulp leading to decomposition. The destruction caused by larvae range from unattractive appearance due to egg laying punctures resulting in reduced marketability and fruit drops leading to diminished yields [4,5]. The management costs in response to the damage are high and in southern Pakistan production of a popular host (guava) was abandoned due to heavy infestations [6]. Regardless of quarantine measures aimed to reduce unintentional introductions of the *Bactrocera* species, their invasions continue to increase [4,5].

Among the *Bactrocera* species that are currently of high interest is the invasive peach fruit fly *Bactrocera zonata* (Saunders) (Diptera: Tephritidae). *Bactrocera zonata*’s invasive nature is linked to it being a strong flier capable of dispersing 25 miles or greater in search of hosts [7], short generation time, high polyphagy, and ability to adapt to different habitats [8]. *Bactrocera zonata* feeds on more than 50 commercial and wild host plants; including peach, guava, mango, apricot, citrus, prickly pear and fig [9,10]. Its host range recently expanded to include some commercially important crops such as eggplant, tomato, apple, loquat, and potato [11]. In the tropical and subtropical regions where it thrives, the availability of its host plants throughout the year favours its proliferation leading to high economic losses in most horticultural regions. The annual financial losses associated with the fruit fly are estimated at USD 200 million in Pakistan [12], EUR 320 million in the Near East and EUR 190 million in Egypt [13]. The amount of fruit damage in Pakistan as a result of *B*. *zonata* infestation is reported to range from 5 to 100% [14]. In addition, it is listed as A1 quarantine pest in the European and Mediterranean Plant Protection Organization (EPPO) countries, affecting availability of lucrative export markets.

*Bactrocera zonata* is native to South and South-East Asia but it has invaded and become established in a number of countries in the Arabian Peninsula, North Africa and some of the Indian Ocean Islands (i.e. Mauritius and Réunion) [8,15,16]. The geographical distribution and abundance of *B*. *zonata* has mainly been attributed to favourable climatic conditions and host availability [4,5,16]. Although *B*. *zonata* is better adapted to tropical and subtropical regions, it is also established in Northern Egypt where temperatures reach freezing point during winter, demonstrating its ability to survive under the Mediterranean climatic conditions [5]. The optimum temperature for adult *B*. *zonata* development is 25°C-30°C whilst egg, larval and pupal survival is highest at 25°C. The upper temperature limit recorded is close to 35°C and none of its stage survives at 12.6°C or less [17]. However, climate change and its associated uncertainties might impact the future global distribution ranges of *B*. *zonata* [18–20]. Globally climate change has altered the 20^th^ century temperatures and its effects are expected to persist in the future [20,21].

Given that *B*. *zonata* continues to invade new areas, there is a need for improved forecasting of potential areas for its invasion and establishment as a mitigating measure. There is a huge deficit in information regarding mapping the potential ecological niche of *B*. *zonata* under current and future climatic conditions accounting for host availability and its likely dispersal patterns. Dispersal is an important factor in insect invasions and plays an important role in determining the potential distribution of *B*. *zonata* [22–24]. To our knowledge, two models of potential geographical distribution of *B*. *zonata* using the CLIMEX model have been published in literature [5,20]. Although they provide important insights into the potential invasive range of *B*. *zonata*, they do not report the impact of host availability and how the fruit fly will potentially disperse in space over time [5,22]. These past attempts to estimating the potential geographical distribution of *B*. *zonata* also did not take into consideration the current invasive location of the pest in Sudan.

In this study we opted to use ecological niche models (ENMs), spatial analysis and spread modeling to determine the potential invasive range of *B*. *zonata*. We include additional occurrence records of *B*. *zonata* for Sudan in our ENM and merge the derived probability of occurrence with host availability data to map areas that are potentially vulnerable. A simple natural dispersal model that mimics the classical spread modeling approaches is developed to determine the potential dispersal pattern of the fruit fly. Ecological niche models link species occurrence and abundance data at known locations with the spatial and environmental properties of those sites to predict the potential distribution of the species across a landscape [25] and are being used extensively [26–28]. One of the ENM algorithms which has been widely employed in modeling potential species distributions in recent years is maximum entropy (MaxEnt) [29,30]. The machine learning algorithm MaxEnt [31] offers a platform to determine areas that are climatically suitable to invasive pests and many such applications are available in literature [32–35]. Coupling ENMs with spatial analysis methods to map areas that are potentially vulnerable and spread modeling strengthens the ability to understand the potential invasive range of *B*. *zonata* [3,36]. Several approaches have been developed to model the dispersal of invasive species ranging from the classical reaction-diffusion models which address short distance dispersal [37–40] to those which focus on long distance and stratified dispersal and other different aspects of dispersal [24,41,42]. The reaction-diffusion models which assume the Gaussian dispersal kernel are used as reference against which other models with different dispersal kernels can be compared [43].

Therefore the objectives of the present study were: (1) To determine areas that are climatically suitable for *B*. *zonata*’s potential establishment under current and future greenhouse gas concentration scenarios in 2050 using the MaxEnt algorithm [44]; (2) To derive host availability data for *B*. *zonata* from the harvested area layer of the Spatial Production Allocation Model (MapSPAM 2005 v3.2) under rainfed and irrigated cropping systems [45]; (3) To combine the MaxEnt output and host availability to generate overall habitat suitability maps for *B*. *zonata*, and (4) To develop a simple spread model for the potential natural dispersal of *B*. *zonata* using the Gaussian probability density function for dispersal kernel.

## Materials and methods

### *Bactrocera zonata* occurrence records

**Fig 1:**
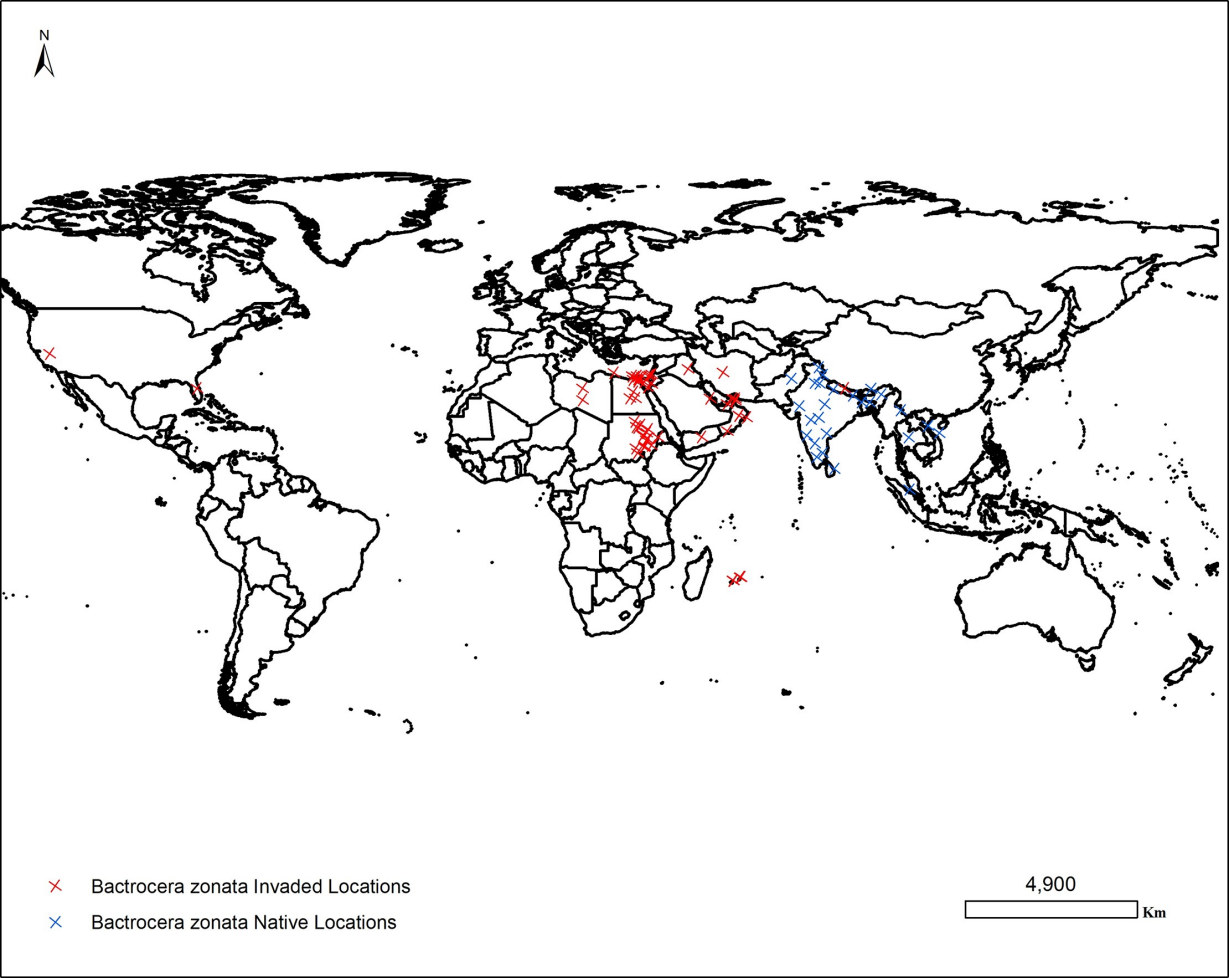
The updated distribution map of the native and invaded occurrence records for *Bactrocera Zonata* collected from the Centre for Agriculture and Bioscience International (CABI) Invasive Species Compendium datasheet number 17694 (n = 37) [8], the Global Biodiversity Information Facility (GBIF) (n = 7) [48] and published articles (n = 7) [20,49–51]. In Sudan, updated georeferenced occurrence records of *B*. *zonata* (n = 57) were obtained from the Agricultural Research Corporation (ARC) of Sudan. “The figure was generated using the QGIS 3.10.2 software (https://qgis.org)”.

Native (n = 40) and invaded (n = 68) occurrence records of confirmed presences of *B*. *zonata* were collected for different countries (**Fig 1**). The utilization of native occurrence records when modeling the potential distribution of invasive species using ENMs significantly improves the precision of the predictive models [20,46,47]. In the present study *B*. *zonata* occurrence records were obtained from the Centre for Agriculture and Bioscience International (CABI) Invasive Species Compendium datasheet number 17694 (n = 37) [8], the Global Biodiversity Information Facility (GBIF) (n = 7) [65] and published articles (n = 7) [20,49–51]. In Sudan, updated georeferenced occurrence records of *B*. *zonata* (n = 57) were obtained from the Agricultural Research Corporation (ARC) of Sudan within a framework of a Department for International Development (DFID) funded project. The presence of *B*. *zonata* in Sudan was monitored during the period 14 January 2014 to 28 April 2016 using methyl eugenol-baited traps to attract and kill fruit fly species in mango, guava, banana, date palm and citrus orchards. The confirmed presences of *B*. *zonata* occurrence with geographical coordinates collected during this period in Sudan were incorporated in the present study.

In preparation for the MaxEnt modeling, duplicate occurrence records were removed, and in instances where georeferenced occurrence points were not provided, but place names given we geo-coded the points based on the place names using Google Earth Pro software version 7.3.2. Although these coordinate points were not the exact locations of the *B*. *zonata’s* records, they provided representative conditions of the sampling sites. The occurrence records were plotted on a map and visually inspected for obvious errors. To minimize spatial autocorrelation, the location records were spatially filtered specifying a minimum distance of 10 km. A similar method was employed in a study to determine the global potential distribution of *Bactrocera carambolae* in Brazil [33]. In other previous studies, models fitted with filtered occurrence records resulted in lower overfitting and had better performance [52,53]. In our study spatial filtering yielded georeferenced occurrence records (n = 74) used in developing the MaxEnt models as some (n = 34) were discarded for being autocorrelated with close by records.

### *Bactrocera zonata* host plant availability

The Centre For Agriculture and Biosciences International (CABI) Invasive Species Compendium datasheet number 17694 [8] was used to extract information on the names of crops considered as important host plants for *B*. *zonata*. These host crop names were used in the subsequent analysis to obtain the spatial extent of the important host plants for *B*. *zonata*, from the Spatial Production Allocation Model (MapSPAM 2005 v3.2) database. MapSPAM 2005 datasets are global gridded maps of crop distribution estimations at 10km x 10km spatial resolution. The maps were calculated according to four variables: actual area where crops are being grown (physical area), harvested area, production and yield for 42 different crops under both rainfed and irrigated production systems. In this study, important host plants for *B*. *zonata* were categorized under four broad classes: tropical fruits, temperate fruits, vegetables, and banana based on the MapSPAM model (**S1 Table**).

Our decision to use MapSPAM products was motivated by an earlier study to determine cropping distributions of the spotted stemborer *Chilo partellus* (Swinhoe) popular host plants in order to assess its potential to invade the areas where they are grown [54]. In the present study, areas where the different *B*. *zonata* host crops are currently grown were aggregated at global level and were used as a proxy for host availability. This involved accessing the harvested area layer (in hectares) of the MapSPAM database to download maps of areas where *B*. *zonata* host crops are being grown. A total of four maps for the different classes of *B*. *zonata* host crops (tropical fruits, temperate fruits, vegetables, and banana) were downloaded. A raster overlay analysis was implemented using ArcGIS version 10.3.1 to combine the maps into an aggregate map which represented areas where hosts are available. The MapSPAM datasets were resampled to 1km x 1km grid cell size using a bilinear interpolation technique in ArcGIS 10.3.1 to match the resolution of the bioclimatic variables for subsequent analysis [55]. The bilinear interpolation method is highly suitable for continuous data like the MapSPAM harvested area dataset.

### Climatic data and variable selection

A set of 19 bioclimatic variables at a spatial resolution of 1 km x 1km freely downloadable from the Worldclim platform (www.worldclim.org) [56], were used as potential predictor variables for modeling the climatic suitability of *B*. *zonata* (**Table 1**) in MaxEnt under current (1950–2000) and future (2041–2060) climatic scenarios. The Worldclim bioclimatic variables were derived by interpolating using a splining technique monthly temperature and precipitation data collected from weather stations across the world. These variables reflect various aspects of temperature, precipitation and seasonality and are important for modeling potential species ecological niches [57,58].

**Table 1:** Worldclim bioclimatic variables used as potential predictor variables in the MaxEnt models [56]. The variables in bold were used in the final models of *Bactrocera zonata’* climatic suitability after eliminating the highly correlated ones.

Bioclimatic variables	Description	Units
Bio 1	Annual mean temperature	°C
Bio 2	Mean diurnal range (mean of monthly (max temp—min temp))	°C
Bio 3	Isothermality (Bio2/Bio7) (* 100)	°C
**Bio 4**	Temperature seasonality (standard deviation *100)	°**C**
Bio 5	Max temperature of warmest month	°C
Bio 6	Min temperature of coldest month	°C
Bio 7	Temperature annual range (Bio5-Bio6)	°C
Bio 8	Mean temperature of wettest quarter	°C
**Bio 9**	**Mean temperature of driest quarter**	°**C**
Bio 10	Mean temperature of warmest quarter	°C
**Bio 11**	**Mean temperature of coldest quarter**	°**C**
Bio 12	Annual precipitation	mm
**Bio 13**	**Precipitation of wettest month**	**mm**
**Bio 14**	**Precipitation of driest month**	**mm**
**Bio 15**	**Precipitation seasonality (coefficient of variation)**	**mm**
Bio 16	Precipitation of wettest quarter	mm
Bio 17	Precipitation of driest quarter	mm
**Bio 18**	**Precipitation of warmest quarter**	**mm**
**Bio 19**	**Precipitation of coldest quarter**	**mm**

**Fig 2:**
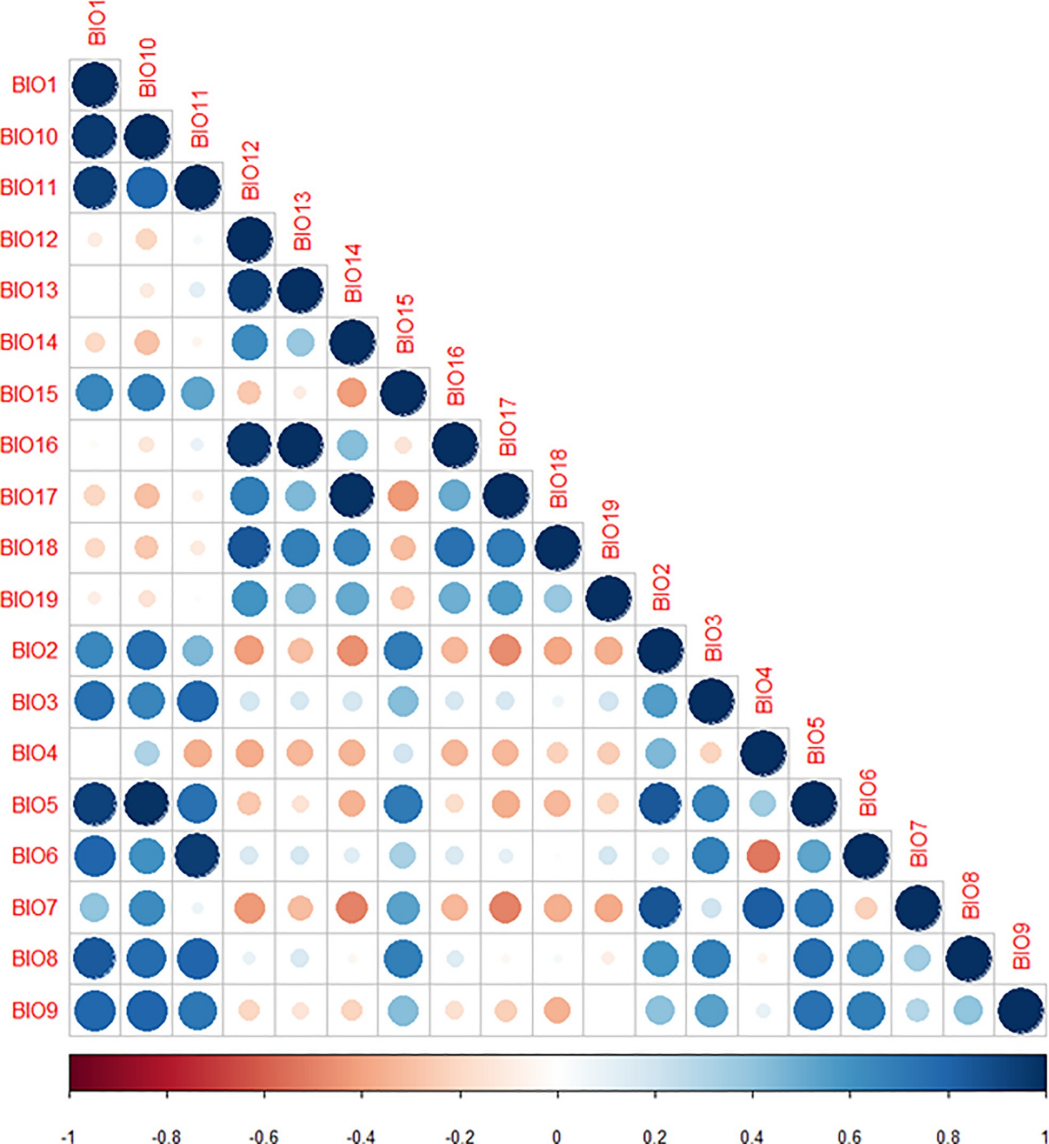
The collinearity matrix for the candidate predictor variables for *Bactrocera zonata*. The collinearity threshold was set at |r|>0.7 according to [61]. Darker shades of blue and red indicate high variable collinearity while lighter shades indicate low collinearity. Similarly, the smaller the circle the lower the correlation value.

To assess the expected multicollinearity between the 19 bioclimatic predictor variables we performed a Pearson’ correlation test between all the potential predictor variables in (**Table 1**). Further, we identified and eliminated variables that were highly correlated using the “Find correlation” function in the Caret package in R using the mean absolute error score [37]. A correlation threshold of |r| > 0.7 was set for variables that could potentially affect our model, and variables that met this criterion were removed from the analysis. The uncorrelated bioclimatic variables were used in MaxEnt to determine the areas climatically suitable for *B*. *zonata* potential establishment. The Pearson correlation graph (**Fig 2**) was generated using the corrplot tool in R software [59,60].

### Possible future climatic scenarios

The Hadley Centre Global Environmental Model version 2-Earth System (HADGEM2-ES) models [62] for four Representative Concentration Pathways (RCPs) 2.6, 4.5, 6.0 and 8.5 were implemented to investigate the impact of climate change on the pest’s distribution in the year 2050. The bioclimatic variables for the different RCPs at a spatial resolution of 1km x 1km were downloaded from the Worldclim platform (www.worldclim.org). The list of the downloaded bioclimatic variables matched the 8 uncorrelated bioclimatic variables determined in the previous section (**Table 1**). The RCPs quantitatively describe concentrations of the greenhouse gases in the atmosphere over time as well as their radiative forcing in the year 2100 [63]. The RCPs are labelled according to their associated radiative forcing in the year 2100 (i.e. 2.6, 4.5, 6.0 and 8.5 Watts per square meter (W/m2) and have carbon dioxide (CO2) concentration levels reaching 421, 538, 670 and 936 ppm, respectively. The projected global mean surface temperature warming for the mid-21^st^ century (2046–2065) compared to the late-20^th^ century (1986–2005) for RCPs 2.6, 4.5, 6.0, 8.5 are 1.0°C, 1.4°C, 1.3°C and 2.0°C respectively [64]. RCP 2.6 represents hard-line mitigation scenarios in literature that limit greenhouse gas concentrations and reduce global radiative forcing by the year 2100 [65]. On the other hand, RCP4.5 and RCP6.0 are intermediate emission scenarios which stabilize after 2100, by applying different strategies and technologies that minimize greenhouse gas emissions [66,67]. Finally, RCP8.5 is considered a high emission scenario with increasing greenhouse gas emissions overtime and the associated increases in global temperatures [65,68]. To review a wide range of possibilities of predicted changes in the potential climatic suitability of the fruit fly we used all four RCPs.

### *Bactrocera zonata* climatic suitability modeling approach

The machine learning algorithm MaxEnt version 3.4.1 [31] was used to predict the areas suitable for *B*. *zonata* invasion based on the selected bioclimatic variables and spatially filtered occurrence records. We chose MaxEnt because it has been widely applied to model potential species distributions with small number of presence-only occurrence data [30,69]. It also resulted in better predictions when its performance was compared with other presence-only methods [70,71]. Evidence from previous studies indicate that using MaxEnt default settings especially with small sample sizes compromises the quality of the predictive model, often resulting in overfitting [72–74]. In order to reduce overfitting in our models, parameter settings were tuned or smoothed as opposed to implementing the default settings in MaxEnt as suggested by earlier studies [72,73]. The performance of MaxEnt largely depends on the choice of feature types and regularization, it was important to modify these parameters for optimal models. Feature types which are allowed shapes of the response curves for the different model covariates allow complex relationships. In our study we tested different combinations of feature classes and regularization to determine the most suitable for modeling *B*. *zonata* and opted to use the linear, quadratic and hinge feature types. These parameter settings allowed for more complex relationships to be modeled as opposed to using “auto features” (a default setting) which are based on the number of occurrence records. Thereafter, a regularization coefficient of 2 was employed as a penalty to the model to prevent overfitting by limiting the strength of the feature classes selected [75]. This is consistent with previous studies which demonstrated that increasing the regularization by two to four times higher than the default settings would result in models with significantly low overfitting [73].

To test the effect of each of the predictor variables on the climatic suitability models, jack-knife tests were performed, and a logistic output format which provides an estimate of the probability of presence was selected. The outlier observations were removed from the final model by implementing a 10% percentile training presence threshold rule.

The occurrence records were randomly divided into 75% training and 25% test datasets using an inbuilt option in MaxEnt. The 25% independent test dataset were used to assess *B*. *zonata* climatic suitability model performance. The Area Under the curve of the Receiver Operating Characteristic (ROC) [76] was used to assess the performance of the MaxEnt models. The use of AUC as a statistic to assess the discriminatory capacity of ENMs has been widely accepted [20,76]. By default, MaxEnt calculates the AUC which determines how the models distinguished between presence and absence observations, but with presence only data as in our case, the AUC compared presence observation with the pseudoabsence background points. The AUC values range from 0 to 1 where values of 0.5–0.7 indicate low accuracy, values of 0.7–0.9 are usually interpreted as useful for applications and those greater than 0.9 imply high accuracy [77].

For consistency, we used the same modeling approach for both current and future climatic scenarios. The outputs of MaxEnt modeling were imported in a geographical information system (GIS) for further analysis. We reclassified our probability of occurrence maps of *B*. *zonata* into 4 classes based on a suggestion by Abdelaal et al. (2019) [78]. The classes were: (i) not suitable (≤ 0.15), (ii) low suitability (0.16–0.30), (iii) medium suitability (0.31–0.60) and (iv) high suitability (≥ 0.61).

### Determining the overall habitat suitability of *Bactrocera zonata*

In order to map areas that are potentially more vulnerable to *B*. *zonata* invasion, climatic suitability derived from the MaxEnt models was merged with host availability extracted from the MapSPAM database using Eq 1. This complemented the results of MaxEnt to get potential climatically suitable areas where *B*. *zonata* could potentially thrive due to hosts being available. The necessity to define areas of potential establishment of invasive species defined by favourable climate and host availability before applying spread models was emphasized before [24]. The datasets were normalized to a common scale of 0 to 1 for easy comparison.

$$S=\frac{n(cs)+n(h)}{2}$$ (1)

Where:

*S* is the overall habitat suitability of an area to *B*. *zonata*, *cs* is the climatic suitability score raster, *h* the host availability raster and *n* is the normalization function. The normalized sum of *cs* and *h* was divided by 2 to ensure that *S* remains in the range of 0 to 1. The normalization was of the form:

$$n=\frac{\left(x-min\left(x\right)\right)}{\max(x)-min\left(x\right)}$$ (2)

Where:

*n* is the normalized output raster, *x* denotes the numerical values in the original raster, min(*x*) and max(*x*) are the minimum and maximum numerical values in the original raster. Numerical values in the range min(*x*) and max(*x*) were rescaled to the range of 0 to 1 in the output raster. The overall habitat suitability of *B*. *zonata* was calculated under current and four future climatic scenarios (RCP2.6, 4.5, 6.0 and 8.5) for the year 2050 to review the potential impact of climate change on the pest’s distribution and to generate relevant maps for potential risk assessment.

The spatial analysis was done in a GIS environment using ArcGIS 10.3.1 and the administrative boundary shapefiles used were acquired from the Natural Earth datasets (http://www.naturalearthdata.com/).

### Simple natural spread model

We developed a simple model to hypothetically model the short distance dispersal of *B*. *zonata* by natural means using the Gaussian probability density function to estimate infestation probability. The following assumptions were considered in building the model: (1) that the host plants of *B*. *zonata* were available throughout the year; (2) the current occurrence records of *B*. *zonata* were the potential source of infestations to its surrounding areas; (3) there were no major barriers limiting the spread of the pest, and that its probability of spread occurred equally in all directions from the source locations; (4) the pest spreads naturally by flying from one location to another in search of suitable host plants; and (5) the pest spreads from its source location with infestation probability decreasing as a function of distance following a normal curve. The following equation for the Gaussian probability density function (3) was used.

$$f(x)=\frac{1}{\sqrt{2\pi }\sigma }e^{-\frac{{\left(x-\mu \right)}^{2}}{{2\sigma }^{2}}}$$ (3)

Where:

μ is the mean of the distribution and σ is its standard deviation. The variance of the distribution is σ^2^.

Dispersal is an important factor in insect invasions and plays an important role in determining their potential distribution including that of *B*. *zonata* [22–24]. *Bactrocera zonata* has the capability of dispersing locally reaching distances of up to 25 miles (40.2 km) [7,8]. Our model uses the known occurrence records of *B*. *zonata* as basis to develop several Gaussian functions centred on each location. Each Gaussian function has a height of 1, a mean of 0 and value (density of fruit fly) decreasing with distance in relation to the width or standard deviation.

In general, the Gaussian probability density functions depict the potential natural dispersal of the fruit fly from one occurrence record site to surrounding locations and the decreased trends mimic inertia to reach far locations. Finally, Gaussian probability density functions were multiplied with the overall habitat suitability distribution raster generated in the previous section to determine the potential natural spread of *B*. *zonata*. The values of the current overall habitat suitability were multiplied by the distance values of the Gaussian functions of closest record locations. A similar method was applied to determine the geographical reachability of the invasive pufferfish in the Mediterranean Sea [36]. This yielded a model which gives the potential natural dispersal of *B*. *zonata* represented by Eq 4

$$l=\frac{\left(k_{1}n(cs)+k_{2}n(h)\right)}{k_{1}+k_{2}}c_{1}e^{-c_{2}x^{2}}$$ (4)

Where:

*l* is the likelihood of natural dispersal of *B*. *zonata*, *x* is the distance from the source locations, *k*_1_ and *k*_2_ are weighting constants, *cs* is the climatic suitability score raster, *h* the host availability raster (in hectares) and *n* is the normalization function. Dividing with weighting constants *k*_1_ and *k*_2_ ensures the normalized sums remain in the range of 0 to 1. Constants *c*_*1*_ and *c*_*2*_ are the controlling parameters for the applied Gaussian probability density function and these were to be estimated.

In our natural dispersal model, *n(cs)* and *n(h)* were given equal weights because they carried equally important information, hence the values of *k*_1_ and *k*_2_ were estimated at 0.5 each. Since the dispersal distances of *B*. *zonata* were assumed to be approximately normally distributed, the values of parameters *c*_1_ and *c*_2_ were estimated as follows: the 3σ for *B*. *zonata* spread was considered as its maximum natural dispersal capacity recorded in literature (40.2 km), giving a σ value of 13.4. Accordingly, the value of *c*_*2*_ was estimated at 0.003 and *c*_*1*_ value was 0.03 based on the Gaussian probability density function. Hence the final model yielded was given by Eq (5)

$$l=\frac{\left(k_{1}n(cs)+k_{2}n(h)\right)}{k_{1}+k_{2}}0.03e^{{-0.003x}^{2}}$$ (5)

Where *l* is the potential natural dispersal, *x* is the distance from the *B*. *zonata* source locations, *n(cs)* is the normalized climatic suitability of *B*. *zonata*, *n(h)* is the normalized host availability, *k*_1_ and *k*_2_ are the weighting constants. Gaussian dispersal kernels have been used for more than half a century [37,38] and they adequately represent the results of short-distance dispersal (diffusion) [43]. They capture the fundamental distance-decay principle of ecology and geography hence their use in developing classical biogeography and spatial dynamics theories. They have been used to model the dispersal of horse-chestnut leaf miner *Cameraria ohridella* in Germany and recently to determine the invasion pattern of a *Lagocephalus sceleratus* (Gmelin) in the Mediterranean Sea [36,79].

The minimum and maximum values from the host availability raster were extracted and used for normalizing the dataset on a scale of 0 to 1. The minimum area under which *B*. *zonata’* host plants are being grown in hectares according to the MapSPAM dataset was 0 and the maximum was 13579.3 hectares. The minimum and maximum values for the climatic suitability models extracted from the MaxEnt model outputs were used to normalize the dataset as shown in **Table 2**.

**Table 2:** The minimum and maximum values of the probability of an area being climatically suitable for *Bactrocera zonata* predicted by the five models under different climatic scenarios ran in MaxEnt.

Climate scenario	Minimum value	Maximum value
**Current**	0.000000037	0.99964601
**RCP2.6**	0.000000000	0.97142601
**RCP4.5**	0.000000051	0.99967700
**RCP6.0**	0.000000061	0.99944902
**RCP8.5**	0.000000040	0.99965697

The model was run under current climatic conditions for one generations of *B*. *zonata* (approx. 46 days), it is known to have between 7 and 9 generations in a year [20].

**Fig 3:**
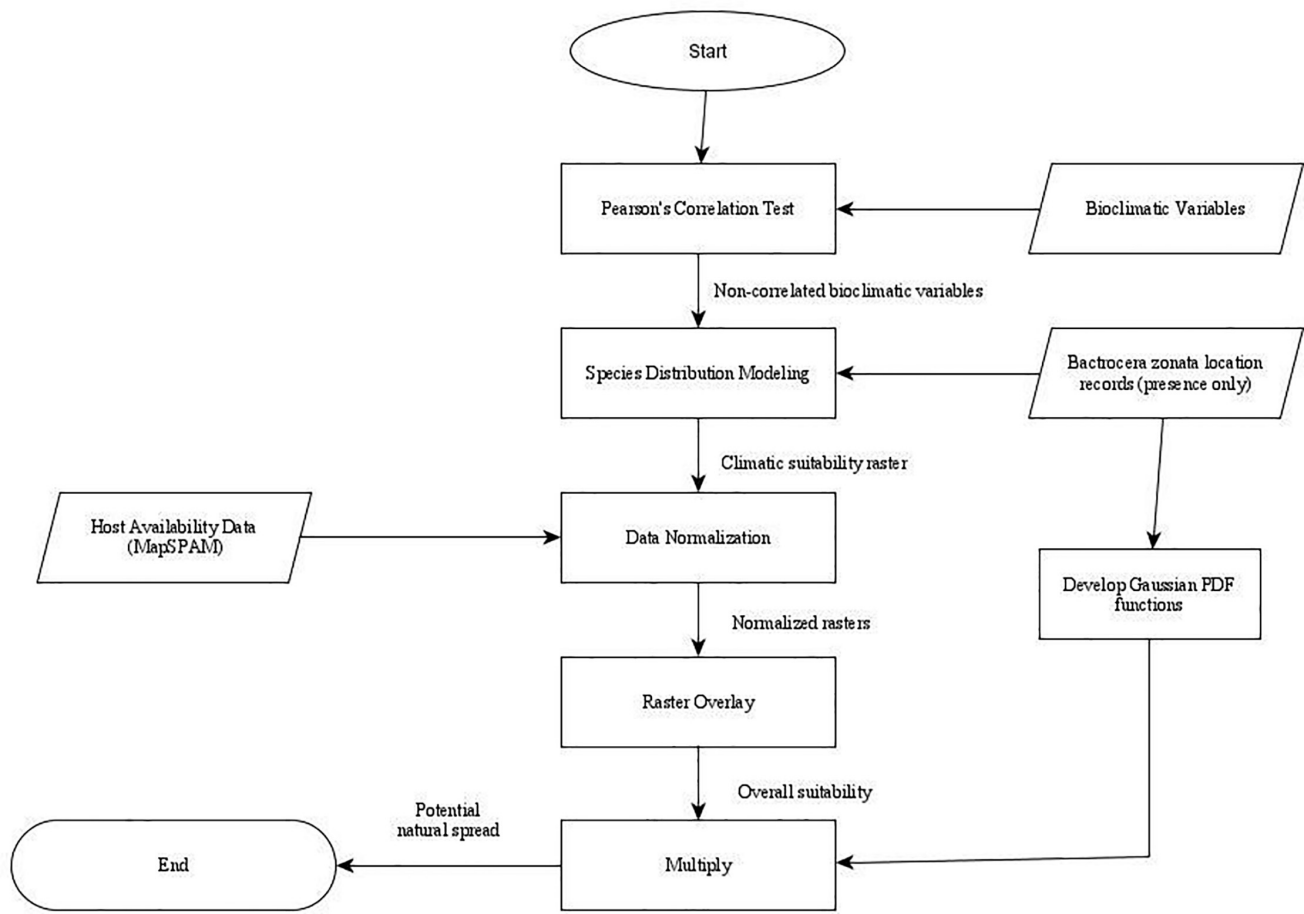
A complete flowchart depicting the datasets and processes employed in the present study.

The methods used in the present study are summarized in a flowchart (**Fig 3**), that shows the datasets and processes employed in building the simple natural dispersal model.

## Results

### *Bactrocera zonata* host availability spatial extent

**Fig 4:**
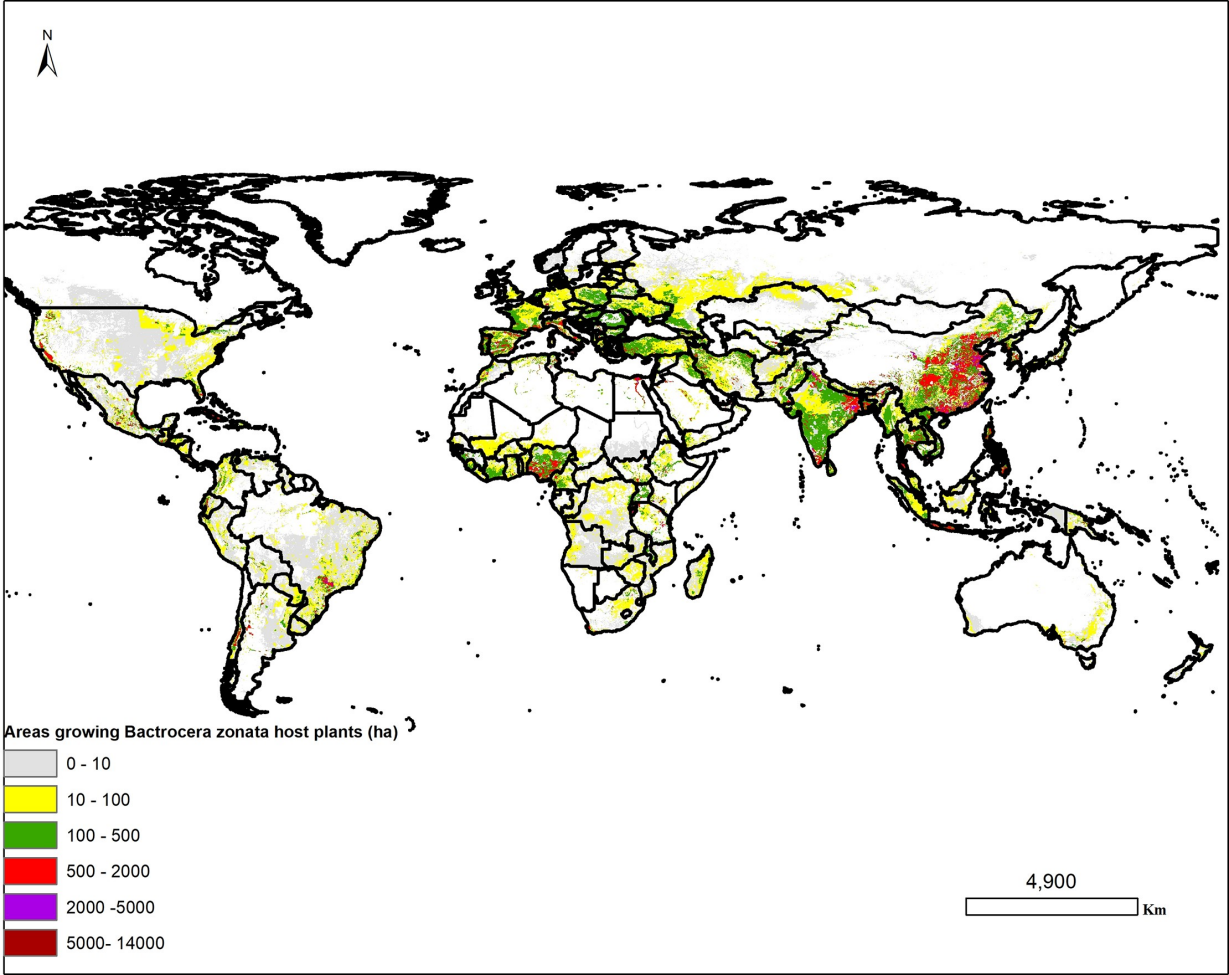
The global distribution of *Bactrocera zonata* host plants under rainfed and irrigated cropping systems in hectares obtained from the Spatial Production Allocation Model (MapSPAM 2005 v3.2) database [45]. “The figure was generated using the QGIS 3.10.2 software (https://qgis.org)”.

The spatial extent of areas growing crops considered as important host plants for *B*. *zonata* in hectares in both tropical, Sub-tropical and temperate regions are presented in **Fig 4.** It can be visually recognised that countries with high *B*. *zonata* host plant cultivation included: North America, Central America, South America, Asia, Europe and much of Africa. These areas with higher harvested areas for the host plants are potentially at higher risk of establishment of the fruit fly.

### Climatic variables importance

The results of the Pearson’ multicollinearity test suggested eight uncorrelated predictor variables (**Table 1**) which were used in the MaxEnt models. Of the eight, three were observed to significantly influence the climatic suitability of *B*. *zonata* under current and future climatic conditions The bioclimatic variables that were regarded as very relevant were “mean temperature of coldest quarter” (Bio11), “precipitation of driest month” (Bio14) and “temperature seasonality (standard deviation *100)” (Bio4). In contrast, precipitation of wettest month (Bio13) had the least contribution. The respective variable contributions in the different models are summarized in the table below (**Table 3**).

**Table 3:** Contribution (%) of the eight bioclimatic variables [56] to the climatic suitability models.

Variable	Current Climate	RCP 2.6	RCP 4.5	RCP 6.0	RCP 8.5
Mean temperature of coldest quarter (Bio11)	35.7	38.9	31.4	31.6	25.9
Precipitation of driest month (Bio14)	31.6	36.5	40.3	39.2	38.3
Temperature seasonality (Bio4)	13.8	0.0	16.6	13.0	17.0
Precipitation of warmest quarter (Bio18)	7.0	13.6	6.4	10.5	7.4
Precipitation seasonality (Bio15)	6.0	0.0	0.9	2.0	4.0
Mean temperature of driest quarter (Bio9)	4.3	7.0	3.1	0.1	6.3
Precipitation of coldest quarter (Bio19)	1.1	2.2	1.9	1.9	1.0
Precipitation of wettest month (Bio13)	0.5	1.8	0.1	1.6	0.1

### Climatic suitability of *Bactrocera zonata* under current and future climate change

**Fig 5:**
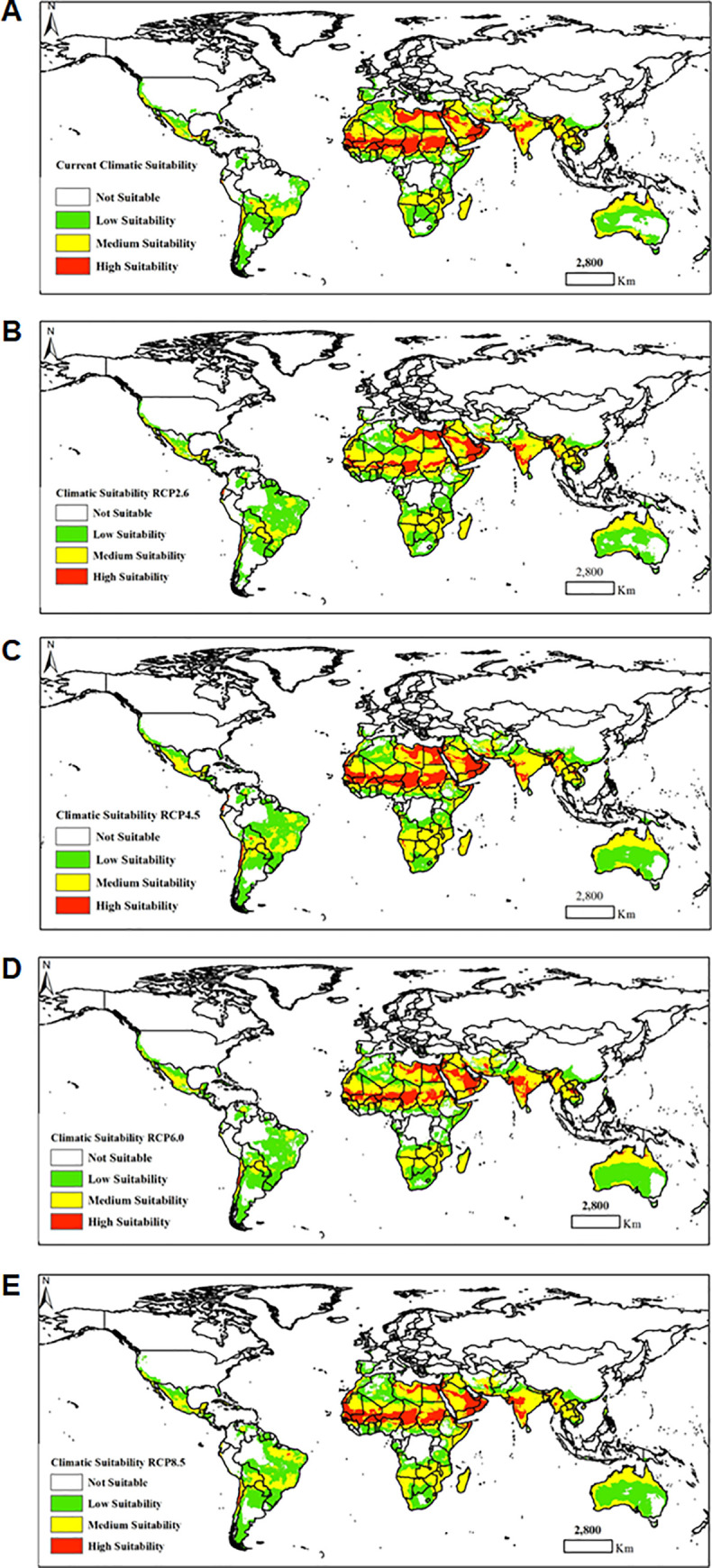
Maps of the climatic suitability of *Bactrocera zonata* under current (A) and four future climate change scenarios [i.e. four representative concentration pathways (RCPs)]–RCPs 2.6 (B), RCPs 4.5 (C), RCPs 6.0 (D) and RCPs 8.0 (E). The climatic suitability classes were: (i) not suitable (≤ 0.15), (ii) low suitability (0.16–0.30), (iii) medium suitability (0.31–0.60) and (iv) high suitability (≥ 0.61). “The figure was generated using the QGIS 3.10.2 software (https://qgis.org)”.

The 5 MaxEnt models using 8 bioclimatic variables exhibited varied results for predicting the areas climatically suitable to *B*. *zonata* establishment under current and future climate scenarios. The results revealed that countries in the Arabian Peninsula (Saudi Arabia, Yemen and Oman), North Africa (Western Sahara, Libya and Egypt), West Africa (Nigeria, Niger, Burkina Faso, Mali, Senegal, Guinea Bissau and Mauritania), Central Africa (Northern Cameroon and Chad), the Horn of Africa (Sudan, Eritrea and to a lesser extent part of Somalia and Ethiopia), Iran, Asia (India, Myanmar, Bangladesh and Bhutan) and South America (Chile and Ecuador) were highly suitable for the potential establishment of *B*. *zonata* (**Fig 5A–5E**). Beside the above-mentioned countries, all the models predicted Madagascar and several countries in Southern Africa and Northern Australia to have medium suitability for the establishment of *B*. *zonata*. However, the model under RCP 6.0 and RCP 8.5 climatic scenarios revealed a significant reduction in areas of high suitability for *B*. *zonata* in Libya compared to the other countries (**Fig 5D and 5E**). Considerable reduction in areas of high suitability was also observed in Yemen and Oman under RCP 6.0 climatic scenario (**Fig 5D**). All the MaxEnt models showed relatively high levels of accuracy in predicting the climatic suitability of *B*. *zonata* as demonstrated by the acceptable accuracies (AUC > 0.9). The AUC values ranged between 0.916 to 0.930 under current and future climatic conditions, respectively (**Table 4**). The models predictive performance indicated that the RCP2.6 MaxEnt model had the highest value of AUC (0.930) and the RCP4.5 model produced the lowest AUC value (0.916) (**Table 4**).

**Table 4:** The AUC values for the five *Bactrocera zonata* climatic suitability models run in MaxEnt.

Climatic Scenario	AUC Value
Current Climate	0.925
RCP 2.6	0.930
RCP 4.5	0.916
RCP 6.0	0.929
RCP 8.5	0.919

### Overall habitat suitability based on aggregated climatic suitability and host availability

**Fig 6:**
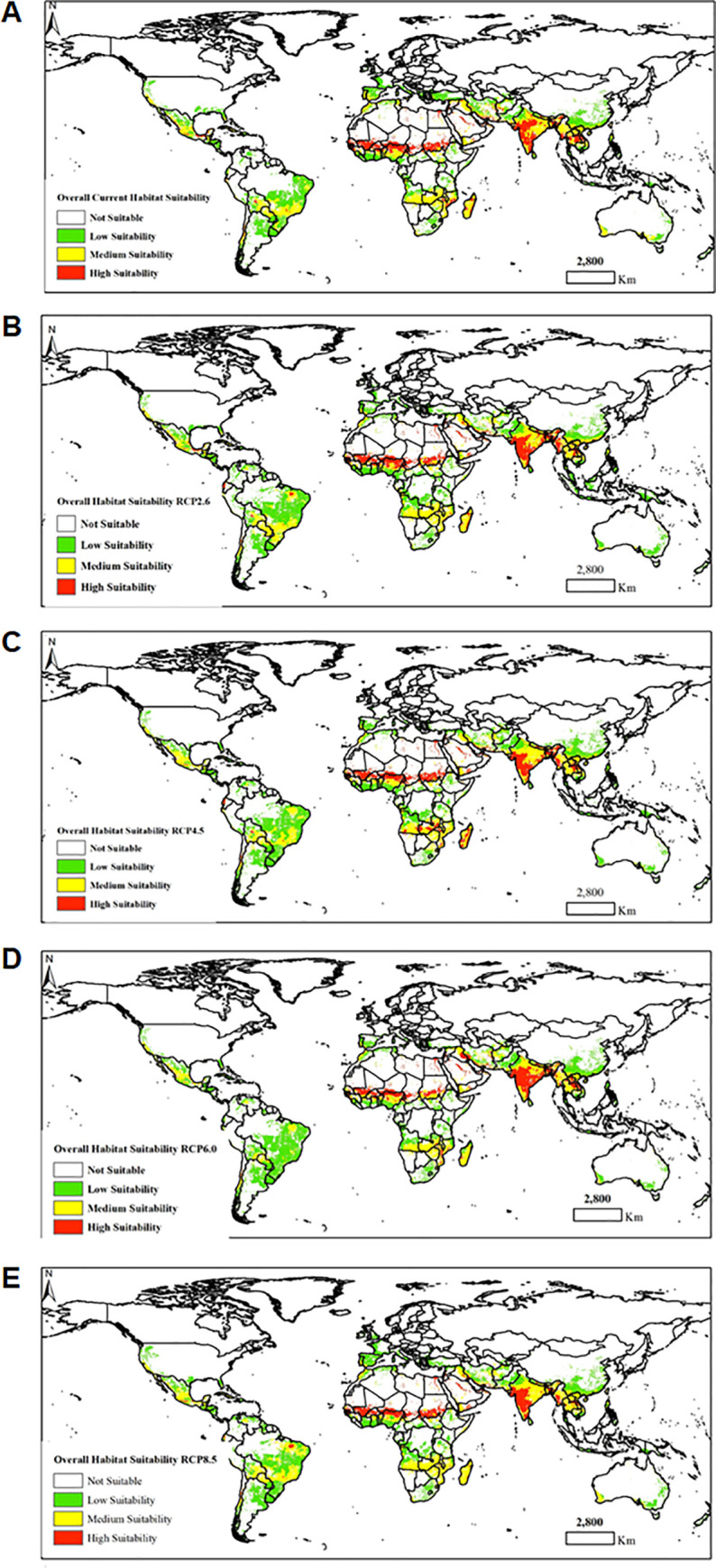
The overall habitat suitability of *Bactrocera zonata* under current (A) and four future climate change scenarios [i.e. four representative concentration pathways (RCPs)]–RCPs 2.6 (B), RCPs 4.5 (C), RCPs 6.0 (D) and RCPs 8.0 (E). These were obtained by merging the normalised climatic suitability with normalised host availability. The overall habitat suitability classes were: (i) not suitable (≤ 0.15), (ii) low suitability (0.16–0.30), (iii) medium suitability (0.31–0.60) and (iv) high suitability (≥ 0.61). “The figure was generated using the QGIS 3.10.2 software (https://qgis.org)”.

The overall habitat suitability of *B*. *zonata* merged climatic suitability and areas growing important host plants for the fruit fly and estimated the areas that were vulnerable to its invasion, the results are presented in **Fig 6A–6E**. We noted some variability in the overall habitat suitability of *B*. *zonata* globally. The merged distribution indicated highly vulnerable areas in Asia (India, Bangladesh, Burma, Thailand, and Laos under future climatic conditions (RCP2.6, RCP 4.5, RCP6.0 and RCP8.5). In Madagascar, Angola, Mozambique, and Zambia suitable habitats were detected under RCP4.5. In Libya, Egypt, Sudan, Chad, Niger, Mali, Mauritania, Western Sahara we predicted areas less vulnerable to the potential establishment of *B*. *zonata*.

### Potential natural dispersal of *Bactrocera zonata*

**Fig 7:**
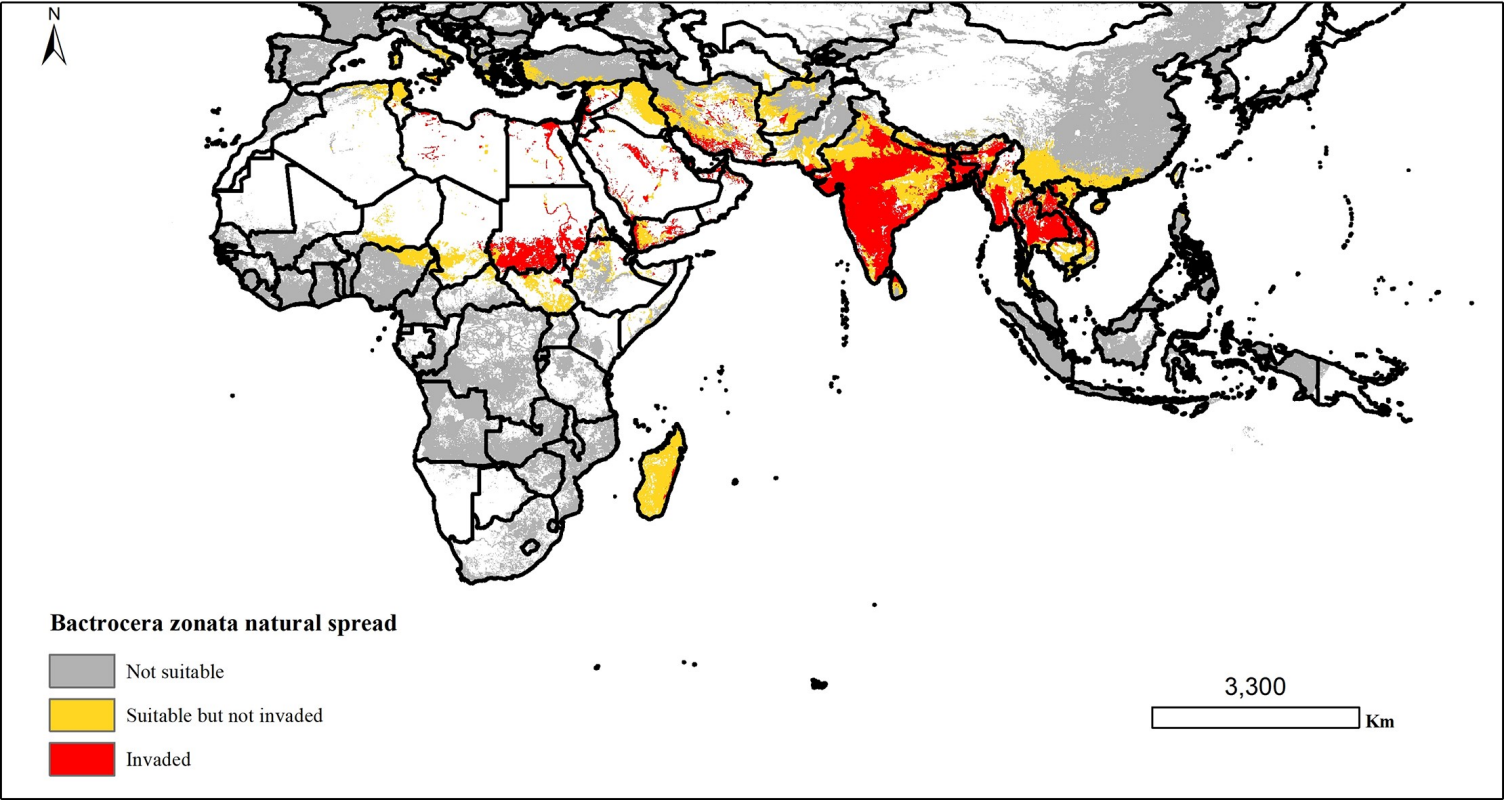
Predictions of the potential natural dispersal of *Bactrocera zonata* based on the simple spread model developed. The model was run for current climatic conditions for one generation of *Bactrocera zonata* (about 46 days), it is known to have between 7 and 9 generations in a year [20]. “The figure was generated using the QGIS 3.10.2 software (https://qgis.org)”.

The potential spread model developed for *B*. *zonata* demonstrated a pattern of dispersal more restricted to areas surrounding the source locations (**Fig 7**). The probability of infestation decreased as a function of distance from the source locations. The model revealed that *B*. *zonata* would spread with high likelihood within Sudan, Egypt, Saudi Arabia, southern parts of Iran, India, Nepal, Bhutan, Bangladesh, Burma, Thailand, Myanmar, and Laos (**Fig 7**).

## Discussion

In this study we used MaxEnt to estimate the ecological niche of *B*. *zonata* under current and four future greenhouse gas concentration scenarios in 2050 to review predictive changes in the potential climatic pest suitability. Climatic suitability derived from MaxEnt was integrated with host availability data to map areas that are potentially vulnerable to invasion by *B*. *zonata*. Further we developed a simple model for *B*. *zonata* dispersal assuming a Gaussian dispersal kernel. The simple dispersal model provides an easy way to map the potential local dispersal of *B*. *zonata* by natural means over time. The resulting potential spread map presents complimentary aspects with respect to short- distance natural spread of the pest. In general, our findings provide information to guide biosecurity agencies at a local level in decision-making and serve as an early warning tool to safeguard against the invasion of *B*. *zonata* into unaffected areas.

According to our knowledge, two studies have investigated the potential climatic suitability of *B*. *zonata* [5,20]. Delrio and Cocco [5] developed the first model of the potential distribution of the peach fruit fly in the Mediterranean basin using CLIMEX (Hearne Scientific, Australia). In their CLIMEX model a set of parameters to describe *B*. *zonata*’s response to moisture and temperature were used to deduce the potential geographical distribution of the fruit fly. Their results suggested the potential establishment of *B*. *zonata* in coastal areas of the Mediterranean region (North Africa) and Near East. In the present study, the predicted potential climatic suitability for *B*. *zonata* was also limited to southern areas of Portugal, Spain, Greece and all the main Mediterranean islands (Balearic Islands, Sardinia, Corsica, Sicily and Crete) similar to that reported by Delrio and Cocco [5].

Furthermore, studies conducted by Ni et al. [20] also using CLIMEX revealed that *B*. *zonata* is expected to potentially establish throughout much of the tropics and subtropics, including some parts of the USA, southern China, south eastern Australia and northern New Zealand under current climatic conditions. Possibilities for expansion of *B*. *zonata* potential ecological niche poleward or northward into colder areas was observed for US, China, New Zealand, and Mediterranean regions under climate change scenarios for the year 2070s [20]. However, our findings indicate a southward spread and potential risk for Sub-Saharan region. Our results are consistent with the recent report from Gezira region in Sudan [11] which indicates a southward spread of *B*. *zonata*.

Contrary to previous studies, the current research work has MaxEnt to predict the potential global climatic suitability of *B*. *zonata* under current and four RCPs for the year 2050 using occurrence data of *B*. *zonata*, and eight bioclimatic variables. MaxEnt was selected because several studies have recommended its use in predicting potential distribution of invasive species [32–35]. The present study took into consideration the recommendations raised by Ni et al. [20] emphasizing the need to incorporate other factors such as host availability and dispersal capacity of *B*. *zonata* in future modeling exercises. These suggestions by Ni et al. [20] have been previously supported by other authors [24], but this has rarely been implemented. In the present study, these factors were considered to gain better understanding of a more precise overall habitat suitability that would allow for the continual survival and proliferation of *B*. *zonata*. In this regard, the Spatial Production Allocation Model (MapSPAM) dataset was used to determine areas where *B*. *zonata* popular host plants are being grown to understand its potential to invade them [45]. These areas where the host plants are being grown were aggregated at global level and merged with the output of MaxEnt using spatial analysis tools. Further, a simple spread model to predict the potential natural dispersal of *B*. *zonata* from source points using the Gaussian dispersal kernel was developed. To improve on the precision of the potential climatic suitability of *B*. *zonata* in MaxEnt, we ensured that our models combined both exotic and native occurrence records of the fruit fly.

Our MaxEnt models were generally more restrictive in predicting the climatic suitability of *B*. *zonata* compared to the results of the previous CLIMEX models, except for north Africa where areas predicted to be optimally suitable were larger than that reported by Ni et al. [20]. Indeed, the model by Ni et al. [20] revealed much larger areas of high climatic suitability, especially across central, eastern, and southern Africa and to a lesser extent the coastal belt of west Africa than in our study. Also, southern and eastern parts of Australia, southern Asia, parts of the Mediterranean areas, China, New Zealand, Spain, Portugal, France, Italy, Madagascar, Central America, South America, and the south-eastern United States were shown to be optimally suitable for possible establishment of *B*. *zonata*, which is contrary to the present study. This could be partially attributed to the differences in the climatic datasets and modeling algorithms used in the studies. There was a strong fit between climatic suitability and known occurrences of the pest. We demonstrated that *B*. *zonata* can potentially establish and become widespread under tropical and subtropical conditions, which agrees with the report by Ni et al. [20]. For all the MaxEnt models we fitted, mean temperature of coldest quarter, precipitation of driest month and temperature seasonality were the most important bioclimatic variables significantly influencing the potential establishment of *B*. *zonata*. Under current and future climate scenarios, optimal suitable areas in Africa were predicted in north (Libya and Egypt), West (Nigeria, Niger, Burkina Faso, Mali, Mauritania, Senegal and Guinea Bissau), East (Sudan), Central Africa (Cameroon, Tchad and Central Africa republic), Arabian Peninsula (Yemen, Oman, United Arab Emirates and Saudi Arabia), Iran, Southern Asia (India, Burma, Bangladesh and Bhutan) and to a lesser extent in South America (Chile). Our model further suggested that under future climatic scenarios, RCP 4.5 and 8.5, there was a significant range expansion of *B*. *zonata* in Western Sahara, and in Southern Africa but with medium climatic suitability. On the contrary, RCP 2.6 showed considerable decrease in *B*. *zonata* range expansion in Central, East and West Africa. All the models under current and future climate change scenarios revealed medium climatic suitability for *B*. *zonata* in Madagascar. However, when the area under production of *B*. *zonata* host plants were taken into consideration the resultant overall habitat suitability map demonstrated less invasion risk for areas that do not currently grow *B*. *zonata*’s host plants. In general, all agricultural areas where *B*. *zonata* host crops are currently grown, and the climatic suitability score is high are considered at higher risk of invasion by the pest.

Furthermore, our models demonstrated that *B*. *zonata* will potentially disperse naturally with decreasing geographical reachability following the normal curve from the present occurrence point locations. Similar use of the Gaussian probability density functions to model the dispersal pattern of an invasive fish species [*Lagocephalus sceleratus* (Gmelin)] to uninvaded areas in the Mediterranean sea from its known locations has been reported [36]. Given the invasive traits of *L*. *sceleratus* its natural spread pattern is likely to be closely related to that of *B*. *zonata*. Gilbert et al. [79] also developed a diffusion model to forecast the potential spread of the horse-chestnut leaf miner *Cameraria ohridella* which was used as a reference against which he compared other leptokurtic models for the pest. Similarly, our simple natural spread model could be used as reference for other leptokurtic models of *B*. *zonata*. Our findings add to the pool of knowledge of previous studies on the potential distribution of invasive species and how they interact with their overall habitat suitability with special emphasis on *B*. *zonata’* natural dispersal.

The simple natural dispersal model developed here complements baseline ENM algorithms which do not support explicit dispersal mechanisms. It supports the concept of short-distance dispersal as an important modeling framework to understand the spread of invasive fruit flies locally. While existing studies acknowledge the limitations of the Gaussian dispersal kernel in realistically modeling human- mediated dispersal through long distance dispersal [43], it adequately represents dispersal by diffusion. While we acknowledge the limitation in our study to model long-distance and stratified dispersal, our model can be used as a reference against which leptokurtic dispersal of *B*. *zonata* can be compared in future. It adequately models short- distance dispersal like the classical spread models and can be applied in local contexts [37,38]. The invasion of *B*. *zonata* will likely involve short and long distance dispersal or a combination of the two which is known as stratified dispersal [1,3,40] resulting in higher rates of spread. This is a complex stochastic spatiotemporal process which involves individual fruit flies dispersing and interacting in a nonlinear matter, and this is better modeled by other approaches like individual based models [3,40].

The predictions for *B*. *zonata* suggest that it could be one of the most serious pests globally due to its ability to tolerate harsh environmental conditions, especially in the northern parts of Africa [5,20], where it is more competitive than several other tephritid species. Libya, Egypt, and Sudan have the highest risk of further invasion and recurrent spread into new areas due to the heterogeneous agricultural landscape of the Mediterranean region, characterized by mixed-fruit and vegetable orchards and therefore deserves special attention. Therefore, the suggestion that cold and dry stress were among the factors that limits *B*. *zonata*’s distributional range in these areas might no longer be applicable. Although to date there are no records of *B*. *zonata* being captured from nationwide monitoring programs in neighbouring countries to Sudan, the potential invasion risk of these countries in the region remains high.

While dispersal is affected by several factors presumably important to *B*. *zonata’*s spread our simple dispersal model did not take into account spatial heterogeneity, dispersal barriers (such as deserts, oceans, mountains) that limit the distances the fruit fly covers in certain directions, natural enemies and human activities such as movement of commodities. While we assume a homogeneous environment, habitat variability plays a key role in the spatial spread of insect pests. In real-world ecosystems the environment is mostly heterogeneous and can greatly affect the rate and pattern of invasive spread. Spreading invasive insects are affected by several natural and human-made obstacles. In addition habitat fragmentation and other spatial arrangements of the favourable habitats have been noted to alter spread patterns of invasive species [2,3].

However, there is scope for improving the delineation of areas that are likely to be invaded as more datasets on *B*. *zonata* become available. Therefore, in future studies and models it will be important to consider some of these factors to improve understanding of their potential role in the distribution of an invasive species such as *B*. *zonata*.

Our findings provide important information to significantly help reformulation of policy decisions, assist government extension officers and farmers to make adaptive agricultural management strategies. This allows for enhanced monitoring and surveillance, and designing of local, regional and national-level phytosanitary and integrated pest management options to limit the spread and reduce impact of *B*. *zonata* in the invaded range. The integration of *B*. *zonata* distribution maps generated under the various climate change scenarios into agricultural landscape management decisions would help to increase crop productivity, secure food production and livelihoods of farmers in affected areas where *B*. *zonata* is likely to expand its ecological niche under climate change. Our modeling results under different future climate change scenarios further enhances awareness on the potential threats of spatial expansion of *B*. *zonata* by the year 2050 and serve as a decision-support tool for early warning signals to guide preparedness for possible invasions.

## Supporting information

S1 Table The list of hosts and classification according to the MapSPAM 2005 v3.2 datasets. The host plants in bold are those that are highly susceptible to *Bactrocera zonata*.
(DOCX)
