# Supplementary material for: Global risk of invasion by Bactrocera zonata: Implications on horticultural crop production under changing climatic conditions
Source: PLoS One. 2020 Dec 23;15(12):e0243047. doi: 10.1371/journal.pone.0243047 (PMC7757907; doi:10.1371/journal.pone.0243047)
Supplement: S1 Table — The host plants in bold are those that are highly susceptible to Bactrocera zonata. (DOCX) [file pone.0243047.s001.docx]

**S1 Table:** The list of hosts and classification according to the MapSPAM 2005 v3.2 datasets. The hosts in bold are the ones important to *Bactrocera zonata.*

| Crop Classification | List of fruits included in MapSpam 2005 v3.2 database |
| --- | --- |
| Tropical Fruits | Papaya *Carica papaya L.* , cashew apple , persimmons *Diospyros virginiana* , date palm *Phoenix dactylifera L.* , pineapples, avocados *Persea americana* , mangoes *Mangifera indica L.* , mangosteens *Garcinia mangostana* , guavas *Psidium guajava L., 1753* , figs *Ficus carica L.* , tangerines *Citrus tangerina* , mandarin *Citrus reticulata*, clementine, oranges *Citrus sinensis* , other melons *Cucumis melo*, citrus fruit *Citrus L.*, grapefruits, lemons, and limes. |
| Temperate Fruits | kiwi, grape, berries, cranberries, blueberries, currants, gooseberries, raspberries, strawberries, pomefruit *Punica granatum*, stonefruit, plums *Prunus subg. Prunus*, sloes, peach *Prunus persica (L.) Batsch*, nectarines *Prunus persica var. nucipersica*, cherries *Prunus Serrulata*, sour cherries, apricots *Prunus armeniaca*, quinces *Cydonia oblonga*, pears *Pyrus communis* and apples *Malus* *domestica* Borkh (Rosaceae) |
| Vegetables | Carobs, okra *Abelmoschus esculentus* , mushrooms, truffes, carrots, turnips, string beans, leguminous vegetables, peas (green), beans (green), leeks, garlic, onion, chillies, peppers, eggplant *Solanum melongena L* (Solanaceae), cucumbers *Cucumis sativus* , gherkins, pumpkins, squash *Cucurbita* , gourds, cauliflower, broccoli, tomatoes Solanum lycopersicum *L* (Solanaceae), spinach, lettuce, chicory, asparagus, artichokes, cabbages and other brassicas |
| Banana | Bananas *Musa acuminata* |
